# Collagen and Silk Fibroin as Promising Candidates for Constructing Catalysts

**DOI:** 10.3390/polym15020375

**Published:** 2023-01-10

**Authors:** Jiankang Chen, Jie Liu, Wen Yang, Ying Pei

**Affiliations:** 1 College of Materials Science and Engineering, Zhengzhou University, Zhengzhou 450001, China; 2 Institute of Physics, Henan Academy of Sciences, Zhengzhou 450046, China

**Keywords:** collagen, silk fibroin, catalysis, carrier, carbonization, porous material

## Abstract

A catalyst determines the mechanism of an organic chemical reaction, thus enabling the commercially viable formation of desired material products. Biopolymers offer new opportunities for the construction of catalysts by virtue of their biocompatibility, environmental benignity, and sustainability, as well as their low cost. Biopolymers are especially useful as carriers and precursors in catalysis application. The employment of biocompatible and biosustainable collagen and silk fibroin materials will revolutionize state-of-the-art electronic devices and systems that currently rely on conventional technologies. In this review, we first consider the ordered hierarchical structure, origin, and processing methods of collagen and silk fibroin. Then, the unique advantages and applicability of collagen and silk fibroin for constructing catalysts are summarized. Moreover, a summary of the state-of-the-art design, fabrication, and application of collagen- and silk fibroin-based catalysts, as well as the application of collagen- and silk-based catalysts, is presented by focusing on their roles as carriers and precursors, respectively. Finally, challenges and prospects are assessed for the construction and development of collagen and silk fibroin-based catalysts.

## 1. Introduction

Catalysts usually play an important role in chemical reactions, acting repeatedly without consumption during the catalytic reaction [1]. The catalyst determines the mechanism of an organic chemical reaction, thus enabling the commercially viable formation of desired material products [2]. Currently, the catalytic industry, which accounts for approximately 30% of the gross domestic product (GDP), promotes the synthesis of more than 90% of chemical substances such as fuels, polymers, textiles, fine chemicals, and pharmaceuticals through catalytic processes [3,4,5]. Therefore, the design and preparation of catalysts have become the focus of extensive attention [6]. An ideal catalyst is preferably recyclable, selective, affordable and ecologically benign, it decreases reaction time, allows avoiding toxic organic solvents, and provides a high product yield [2]. The dispersed active centers in the homogeneous catalyst are the main factors that determine the high activity of the reaction. The applicability of the homogeneous catalyst is limited because of disadvantages such as corrosion of the metal reactor, low stability, fast deactivation, and difficulty in separating and recycling [7]. Loading the active components on a carrier has been proven as an effective method for fabricating heterogeneous catalysts. This method not only makes the recovery of the catalyst easier but also reduces the catalyst cost [8]. The stability, surface qualities, specific surface area, and pore structure of the carrier greatly influence the uniform distribution, availability, shape, size, electrical structure, and support–metal interaction of the active sites [9]. For example, the catalytic activity of the supported metals depends on the particle size distribution, and support–metal interactions of carriers [10]. Inert powders as the common carriers require costly surface modifications and recovery treatments [11].

Biopolymers, including alginate [12,13,14], cellulose [15,16], starch [17,18], and chitosan, have been reported as carriers for immobilizing active catalytic substances. Biopolymers with renewability, high adsorption, and metal-stabilizing properties are better candidates for constructing catalysts than inorganic and organic synthetic materials [19]. The abundant functional groups and adaptable structures stabilize active metals and avoid the aggregation of metal particles [20]. In addition, these biopolymers, derived from biomass with an ordered hierarchical structure, are valuable feedstocks for generating biochar-based catalysts.

Among biopolymers, collagen and silk fibroin as natural proteins exhibit excellent biocompatibility, biodegradability, and low toxicity, making them good candidates for constructing catalysts [21]. Protein, as a component of natural tissues, is a rational choice for various applications. As natural proteins, collagen and silk fibroin are sustainable, economical, and reliable feedstock because of their competitive resource advantages [22]. As an essential component of the extracellular matrix, collagen is the dominant structural protein in animals. In the collagen family, type I collagen accounts for the largest proportion of these variants in most biological tissues, including skin, bone, tendon, cartilage, cornea, and blood vessels [23]. Each year, various collagen-containing wastes and byproducts are generated from the leather and food industries, providing sufficient resources for utilizing biological collagen in new value-added materials [24]. For example, the seafood processing industry generates a large amount of collagen-containing biowaste yearly, accounting for about 25% of the total production. In leather processing, more than 700 kg of collagen-containing biowaste is generated when 1000 kg of raw hides is converted [25]. Currently, animal wastes such as animal bones, fish scales, and discarded cowhides are widely used in developing heterogeneous catalysts. [26]. For centuries, Bombyx mori silkworm silk fibroin has been used as a high-end textile fiber. Silk fibroin of the silkworm, a commonly available natural protein, is exploited as a result of its excellent properties of elasticity, biocompatibility, mechanical strength, and controllable biodegradability. Sericulture has a long history of more than 5000 years in China. Cocoon production in China is about 500,000 tons per year, accounting for 70% of the total world production [27,28].

In this review, the ordered hierarchical structure and processing methods of collagen and silk fibroin are introduced. Then, the unique advantages and applicability of collagen and silk fibroin for constructing catalysts are summarized. In addition, an important summary of the state-of-the-art design and fabrication of collagen and silk fibroin-based catalysts is presented. Finally, challenges and prospects are assessed for the construction and development of collagen- and silk fibroin-based catalysts.

## 2. Review Methodology

**Figure 1:**
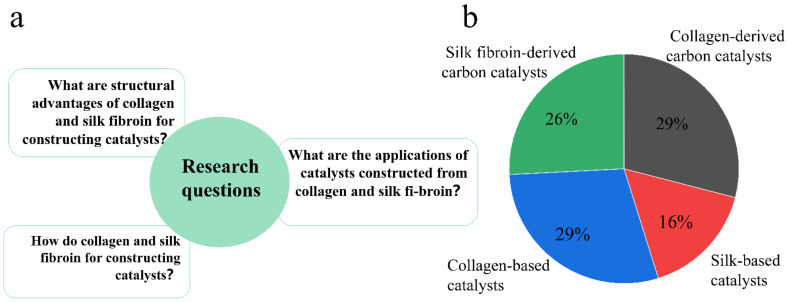
(**a**) Review of the issues to be discussed. (**b**) Proportion of papers on collagen and silk as catalyst carriers and precursor, respectively.

This review aims to introduce the latest progress in the field of collagen- and silk fibroin-based catalysts. It also provides a summary of past years of research in this field. At the beginning of the review, we define the following research questions (Figure 1a). To guarantee the completeness and accuracy of this review, the search strings used allowed for correct coverage of the addressed topics. The literature search was conducted with the specific keywords ‘collagen’, ‘silk fibroin’, ‘catalysis’, ‘carrier’, ‘carbonization’ and combinations of the latter, using advanced search in the databases. The following databases were used:

- Scilit (scilit.net).
- Web of Science (scholar.google.com).
- Scopus (scopus.com).
- Wiley (onlinelibrary.wiley.com).
- Web of Science (webofknowledge.com).

We selected published journals and books in English. Then, identified and selected the most suitable papers, saved typical literature, and selected articles relevant to the research topic. The literature search did not set a time frame, and the final literature retrieval date was September 2022. We selected 85 research papers involving collagen- and silk for constructing catalysts (62 original articles, 23 review articles). Finally, we sorted and classified the selected literatures (Figure 1b). The number of literatures that used collagen/or silk as catalyst precursors was higher than the number of literatures that directly used them as catalyst carriers. Their hierarchical structure makes them advantageous as carbon-material precursors.

## 3. The Structural Advantages of Collagen and Silk Fibroin for Constructing Catalysts

**Figure 2:**
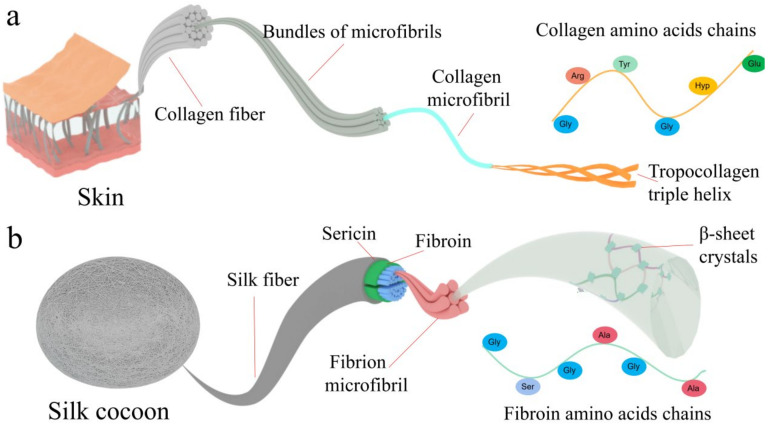
Hierarchical structures of collagen and silk fibroin. (**a**) Schematic diagram of skin, collagen fiber, triple helix, and molecular structure. (**b**) Schematic diagram of cocoon, silk fiber, sheet, fibroin, and molecular structure.

Both collagen and silk fibroin have an ordered hierarchical structure from molecular-scale, mesoscale, to macroscale. This multilevel structure is one of the advantages of using collagen and filaggrin for constructing catalysts. As shown in Figure 2a, collagen molecules with a length of about 280 nm and a diameter of 1.4~1.5 nm consist of three left-handed polypeptide chains [29]. Various amino acids, including glycine (Gly), alanine, glutamate, arginine, L-cysteine, tryptophan, tyrosine, and other essential amino acids are located on collagen polypeptide chains. Each polypeptide chain is composed of a series of Gly-X-Y repeats, an N-terminus and a C-terminus. The Gly-X-Y repeat is the most common amino acid sequence, where X and Y are occupied mainly by proline and hydroxyproline acid [30]. Three peptide chains are entangled with each other to form a right-handed triple-helical collagen called tropocollagen. Tropocollagen subunits assemble into larger arrays and then further aggregate into collagen microfibrils. Multiple bundles of microfibrils with the same or similar orientation are aggregated and bundled into a collagen fiber at the higher level [31]. Collagen fibers and their bundles are standard structural units in many tissues (e.g., skin, tendon, and bone), thus forming a more complex and diverse macrostructure such as the three-dimensional (3D) network composed of collagen fibers in the skin and the orientation structure with collagen fiber arrays in tendons. Multiple forces stabilize these hierarchical structures of collagen including intramolecular and intermolecular hydrogen bonding, electrostatic actions, the van der Waals force, hydrophobic actions, aldehyde-amine cross-linking, and aldol-histidine [32]. As shown in Figure 2b, 18 amino acids including alanine, glycine, tyrosine, and serine exist in silk fibroin polypeptide chains [33]. The aggregations of polypeptide chains form highly oriented antiparallel *β*-sheet crystals, helices, and a random coil structure. These aggregation structures of silk fibroin further assemble into nanofibrils and their bundles.

Based on the above multi-hierarchical structure characteristics, we can summarize the advantages of collagen for the construction of catalytic materials. First, the molecular structures of collagen and silk fibroin are rich in C, N, O, and S atoms, which make them potential precursors for producing heteroatom-doped carbon, especially N-doped carbon [34]. The difference in atomic radius, bond length, and electronegativity between doped N and C theoretically causes defects. These structural defects in carbon materials mainly account for the disruption of the symmetrical distribution of charge density, which results in the localization of electrons at specific locations and the formation of new active sites [35]. In addition, heteroatoms with strong coordination are used to anchor metal ions or metal nanoparticles as catalytic centers. These ligand-type interactions maximize the dispersity of metal nanoparticles, prevent aggregation, and promote carbon–metal interactions, establishing an efficient metal–ligand electron transfer between metal nanoparticles and carbon [36]. The introduction of heteroatoms in the carbon structure enhances the π-bonds, thereby improving the stability and electron transfer rate and the performance [37]. For N-doped carbons, the electronegativity of N induces positive charges on adjacent C atoms, which plays a crucial role in boosting oxygen reduction reaction (ORR) catalytic activity [35,36,38]. Ying-Hui Lee [39] demonstrated that the N-doped structure in N-doped nano-sheets generated by the carbonized collagen could serve as the active site of the ORR. Runqing Lu [40] reported that nano-nickel-cobalt-iron (NiCoFe) alloys were embedded on conductive boron (B) and N co-doped/silk-derived carbon aerogels as oxygen evolution reaction (OER) electrocatalysts. The doping of N and B improved the hydroxide adsorption capacity of the electrocatalyst and further promoted the species-deprotonation and O_2_ generation.

Second, amino acids of collagen and silk exhibit special spatial effects and strong coordination with metal ions, making them excellent catalyst carriers [41]. The catalytic efficacy of catalysts is improved by enhancing active sites, decreasing particle size [42,43], and enhancing the dispersity of active particles [44]. The active metals (Ag, Cr (III), Fe (III), and Zr (IV) [45,46]) form biological coupling reactions with the hydroxyl, carboxyl, amine, and other functional groups in the protein chains [47,48] and fix them on the collagen fibers. Bi Shi’s research group reported that Fe(III) ions were fixed on collagen fibers to prepare a renewable heterogeneous photocatalytic catalyst for the degradation of Malachite Green MG and Orange II [49,50,51]. Fe forms a stable hydroxyl complex with the -carboxyl group on the collagen surface as an active site.

Third, the structures of collagen and silk fibroin facilitate the synthesis of porous carbon nanomaterials. Compared with powdered or membrane-like catalysts, porous carbon materials have better geometric and heat transfer properties, and therefore are more favorable for liquid or gas phase reactions [52]. In addition, the porosity and high surface area enhance the electron transfer rate during catalysis [53]. For example, N-doped porous carbon was obtained by using animal bone as a precursor, which has an organic carbon skeleton and inorganic constituents [54,55]. Applying natural collagen fibers as precursors, GaoXiao [56] synthesized metal nanoparticle-containing carbon composite aerogel composites that showed greater durability and electrocatalytic ORR performance than the commercial Pt/C catalyst. Silk fiber is a typical natural N-rich material with a fibrous structure, which is transformed into N-doped carbon fibers by a simple heat treatment [57]. Tomoya Iwazaki reported [58] that activated carbon obtained from carbonized silk fibroin showed high catalytic activity for the ORR in a sulfuric acid solution. Moreover, the steam activation of carbonized silk fibroin at 850 °C greatly enhanced the catalytic activity. The highest onset potential of the ORR of silk fibroin-derived activated carbon was 0.84 V at 60 °C. In addition, one-dimensional (1D) S, N co-doped micro/mesoporous carbon materials were obtained by using cocoon silk as a precursor for activated carbons of cocoon silk fibroin with steam [58], KOH [59], KCl [60], or zinc chloride [53].

## 4. The Processing Methods of Collagen and Silk Fibroin

Collagen and silk fibroin have been processed into various types of bulk materials such as hydrogels [61,62,63], sponges [64], and films [65,66] for application in different catalytic reaction scenarios. The bottom-up and top-down approaches are common methods of designing and assembling collagen and silk fibroin. A bottom-up approach is one in which smaller components of molecular dimensions self-assemble in accordance with externally applied driving forces to form larger, more organized systems [67]. This approach has been applied to prepare collagen materials. Collagen can be dissolved in salt solutions [68], ionic liquids [69], acidic solutions [70], and alkaline solutions [71] for further processing. Dan Luo [72] poured collagen type I solution into a dialysis bag, which was then put into a mixed solution containing phosphate-buffered saline (PBS), ruthenium chloride (RuCl_3_), and sodium tripolyphosphate (TTP) in a flask. After a 24 h reaction, the suspension was formed by centrifugation and stirring and then freeze-dried to obtain Ru-doped collagen. Finally, the Ru-doped collagen was pyrolyzed at 800 °C for 2 h to obtain a 3D porous Ru-doped collagen-based carbon scaffold (Ru-CCS). The Ru-CCS had good hydrogen evolution activity with overpotential and Tafel slopes that were comparable to those of Pt/C catalysts. Several chemicals, including chaotropic salts (lithium bromide, calcium chloride/ethanol, and calcium chloride/formic acid), organic solvents (hexafluoroisopropanol (HFIP) and trifluoroacetone), and organic salts (N-methyl morpholine N-oxide and ionic liquids), were utilized to dissolve silk fibers to obtain a silk fibroin solution [73]. For example, the degummed silk fibers were directly dissolved in a calcium chloride/formic acid solution. A simple regenerated silk fibroin (RSF) hydrogel with uniform pore structure and good mechanical properties was obtained by fibroin regeneration in water. As an ideal reusable catalyst, Fe_3_O_4_@RSF hydrogel was further prepared by establishing interactions between the Fe ions and functional groups in silk fibroin [74]. The collagen and silk fibroin materials prepared with this bottom-up approach had a uniform size and orderly arrangement, but the natural hierarchical structure was destroyed during the dissolution process.

The top-down approach refers to the exfoliation of the micro/nanofiber structures from the superstructure of collagen and silk fibroin as building blocks to fabricate bulk materials [75]. Top-down physical methods mainly include microfibers or nanofibers obtained from collagen and fibroin [76]. Collagen micro/nanofibers were obtained by liquid exfoliation methods using NaOH/urea [77], HCl solution [78], high-pressure homogenization [76], grinding, and high-intensity ultrasound [79]. Silk fibroin nanofibers were also prepared by liquid exfoliation of silk fibers in sodium hypochlorite aqueous solution [80], urea/GuHCl [81], and citric acid/choline chloride systems [81,82]. Rui Wang [83] delimed, softened, and cured cowhide and treated it with an aqueous acetic acid solution to remove the non-collagenous components. The cowhide was then dehydrated and dried with absolute ethanol, placed under a vacuum at room temperature for 24 h, then the treated cowhide was ground and sieved (5–10 mesh) to obtain collagen fiber powder. Finally, collagen fibers based on tannic acid fixation and Fe^3+^ (TA-Fe-CF) cross-linking were prepared using collagen fiber powder as a carrier. The hierarchical TA-Fe-CF catalyst exhibited an excellent adsorption capacity in the early stages before the introduction of PMS. Moreover, TA-Fe-CFs improved the utilization efficiency of sulfate radicals in subsequent sulfate radical-based advanced oxidation processes. These collagen and silk fibroin micro/nanofibers have high specific surface area, a great number of functional groups and active sites, which enhance the density and stability of immobilized Fe^3+^.

## 5. Catalysts by Using Collagen and Silk Fibroin as Carriers

Physical loading and chemical bonding are used to manufacture collagen and silk fibroin-based catalysts. The active components of the catalyst rely on the van der Waals force to combine with the carrier in the physical loading approach, which has the disadvantages of weak combination and easy shedding of active components. Chemical bonding connects a homogeneous catalyst with a polymer support via a chemical bond (ion bond or covalent connection). It is the most common way to create catalysts supported by collagen and silk fibroin.

### 5.1. Collagen-Based Catalysts

**Figure 3:**
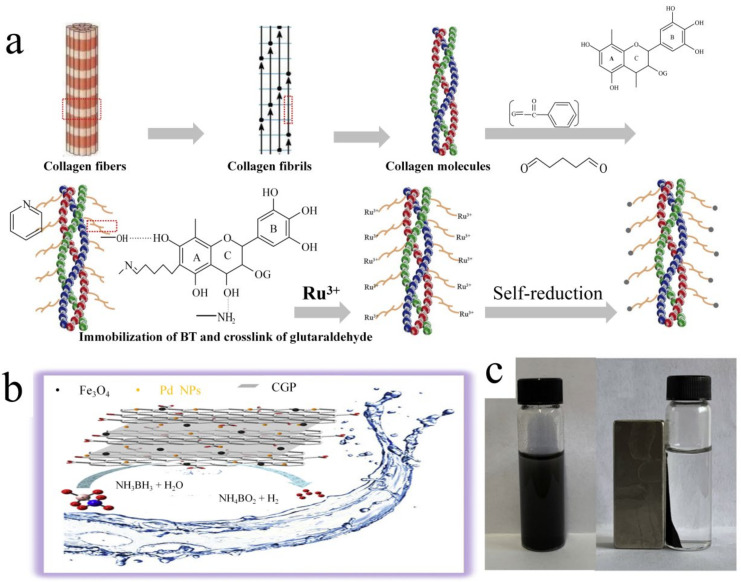
(**a**) Schematic diagram of CF-BT-Ru synthesis. Reprinted from Ref. [91] Copyright (2021) with permission from Elsevier. (**b**) Schematic diagram of Pd/CGP-GO-Fe_3_O_4_ catalyzing hydrogen production from ammonia borane, (**c**) recovery of Pd/CGP-GO-Fe_3_O_4_. Reprinted from Ref. [95]. Copyright (2019) with permission from Elsevier.

As mentioned above, collagen has an affinity with metal ions through interactions between the metal ions and the amino acid groups in collagen, which is beneficial to preparing catalysts. Chemical modifications of collagen were applied to enhance this affinity, as well as to improve its stability. As shown in Table 1, epigallocatechin-3-gallate (EGCG) and bayberry tannin (BT) as cross-linking agents were reacted with collagen to establish stable connections or interactions with metallics. Because of the high concentration of phenolic hydroxyl groups, plant tannins can form multi-point binding with polypeptides, enter the inner space of collagen fibers, and generate cross-linked structures between adjacent polypeptide chains [84]. Under acidic non-oxidative conditions, phenolic hydroxyl groups as hydrogen donors can form polyphenol derivatives with the carbonyl in collagen as a hydrogen receptor, and the free amino groups in collagen as hydrogen donors can form hydrogen bonds with phenolic hydroxyl in the cross-linkers as hydrogen receptors [85,86]. Hydrophobic interactions can occur between the hydrophobic domain of aromatic rings in cross-linkers and the hydrophobic side chains of amino acids in collagen. Under alkaline conditions, they oxidize the phenolic hydroxyl groups in these polyphenol derivatives to quinones, which can react with collagen mainly through a Schiff base reaction and a Michael addition reaction of the quinone with histidine and lysine amino acids and homocysteine mercapto genie cross-linking [87]. In addition, plant tannins have multiple adjacent phenolic hydroxyl groups, showing a special affinity with metal ions, and the metal particles are reduced to form metal nanoparticles, which improves their loading and stability [88]. Hao Wu [89] used natural polyphenol EGCG to cross-link collagen fibers to prepare a new stable heterogeneous palladium (Pd) nanoparticle catalyst. The results indicated that Pd(II) ions were first anchored on the EGCG–collagen fibers by forming a five-member chelating ring with the ortho-phenolic hydroxyl group of the EGCG. The grafting of EGCG significantly enhanced the interaction between collagen fibers and Pd ions and dispersed the Pd nanoparticles to be uniformly distributed on the outer surface of ordered collagen fibers. In moderate environments, these stable Pd(0) nanoparticles may catalyze the hydrogenation of nitrobenzene and its derivatives, demonstrating good activity, selectivity, and reusability as catalysts. Hui Mao [90] grafted black spot tannin onto collagen fibers as a stabilizer and carrier of Pd nanoparticles, then synthesized a recyclable heterogeneous substance and a Pd catalyst (Pd-BT-CF). The Pd nanoparticles were uniformly dispersed on the surface of the catalyst in an orderly fiber state. Pd-BT-CF exhibited excellent reusability and was 100% selective to 1,2,3,4-tetrahydroquinoline during the reaction step. Studies have found that the hydroxyl group of BT-CF has a stabilizing effect on Pd, effectively preventing the agglomeration and shedding of Pd nanoparticles. Recently, Ling Fu [91] reported the preparation of Ru-based catalysts for ammonia borane hydrolysis by using collagen fiber-immobilized BT as a support for metallic Ru (Figure 3a) (CF-BT-Ru). The CF-BT-Ru retained the natural fibrous structure of collagen, and Ru was uniformly dispersed on the fiber surface. The CF-BT-Ru possessed excellent catalytic activity while exhibiting excellent stability and reusability. Wang [92] used the organic–inorganic in situ method to prepare a silver salt/collagen fiber hybrid composite. The surface of the silver salt/collagen fiber hybrid composite is rougher than that of pure collagen fiber because the silver salt particles of 300–500 nm are uniformly dispersed in it, which increases the specific surface area of the collagen fiber. In addition, collagen fibers can reduce the agglomeration of inorganic particles. Under visible light irradiation, the degradation rate of AgCl/collagen fibers to methyl orange can reach 90% within 30 min. In addition to being a carrier for metals and metal ions, collagen is also commonly used as a carrier for metal oxides. Nagaraj [93] designed a photocatalytic nano-bio-sponge based on collagen–titanium dioxide (TiO_2_) nanoparticles for photocatalytic degradation of rhodamine-B (RhB). The TiO_2_ nanoparticles were functionalized with 3-aminopropyltriethoxysilane to stabilize collagen fibers derived from cowhide waste, resulting in a 20 °C improvement in collagen fiber thermal stability compared to pristine collagen fibers. The nano-bio-sponges featured a multi-layered sponge structure with linked pore structures that destroyed more than 95% of the RhB in 130 min. In addition, collagen can be combined with inorganic substances to increase collagen structure diversity and create catalysts with distinct topologies. In 2016, Hossein [94] created an efficient magnetic nano-catalyst Fe_3_O_4_@SiO_2_/collagen for benzimidazole and benzothiazole derivative production. The catalyst exhibited a homogeneous spherical core–shell shape with approximately 90% of the particles in the 26–53 nm size range. The catalyst achieved an excellent yield of 97% during catalysis, and it retained good catalytic activity after four repetitions, indicating its stability.

In recent years, graphene has been favored for its high specific surface area, high charge mobility, and high stability [96]. Graphene oxide (GO) has many functional groups, making it more active than graphene. Its characteristics can be modified by reacting with different functional groups [97,98]. Hang Jia [95] created a tightly packed structure as a Pd carrier using a magnetic collagen powder–GO mixture (CGP-GO-Fe_3_O_4_)_y_. Pd/CGP-GO-Fe_3_O_4_ had excellent catalytic activity for the hydrolysis of borane ammonia (NH_3_BH_3_, AB) because of the numerous functional fragments of collagen and GO that contribute to the dispersion of Pd (Figure 3b). AB hydrolysis had a 36.5 kJ mol^−1^ activation energy and a turnover frequency (TOF) of 27.4 mol_H2_ mol^−1^. Furthermore, the addition of magnetic materials improved the catalyst’s recyclability (Figure 3c), reduced the cost of the catalytic reaction, expanded the catalyst’s application potential, and generated new ideas for collagen-based catalyst modification.

### 5.2. Silk Fibroin-Based Catalysts

**Figure 4:**
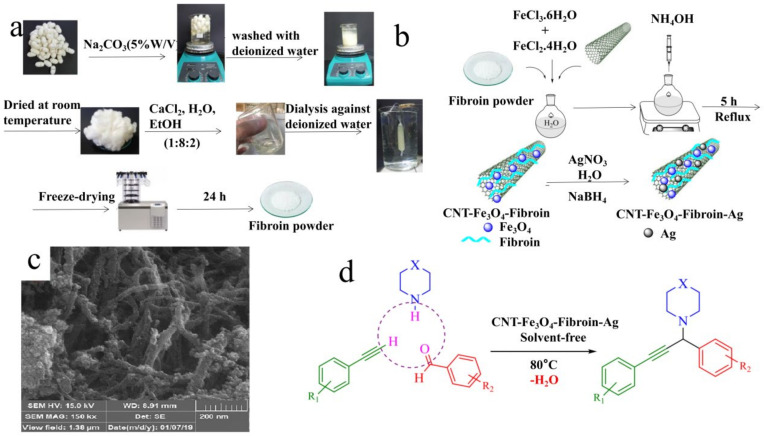
(**a**) Preparation procedure of fibroin powder, (**b**) process for the preparation of CNT–Fe_3_O_4_–fibroin–Ag, (**c**) scanning electron microscopy images of CNT–Fe_3_O_4_–fibroin, (**d**) A3 coupling reaction catalyzed by the CNT–Fe_3_O_4_–fibroin–Ag nanocomposite. Reprinted from Ref. [111] Copyright (2019) with permission from The Royal Society of Chemistry.

Aromatic, amine, hydroxyl, carbon, or sulfur functional groups in silk fibroin form bioconjugates with metals, making proteins suitable carriers for metal-based catalysts. S. Akabori [103] prepared the first silk fibroin-palladium complex by adsorbing palladium chloride on silk fibroin in 1956, resulting in an asymmetric catalyst. It complexed and produced an asymmetric catalytic active center using the stable asymmetric protein structure of silk fibroin, which efficiently catalyzed the selective hydrogenation of various substrates at a pressure of 80–90 MPa and a temperature of 70–80 °C. S. Akabori [104] published research on silk fibroin platinum catalysts in 1961. However, Akabori’s synthesis of silk fibroin–metal composite catalysts required highly acidic conditions, which may lead to the denaturation of silk fibroin. Based on the research of S. Akabori and Zhou, Hironao Sajiki [105,106,107] developed the preparation process. The Pd/fibroin catalyst was made using methanol as a solvent and reducing agent and automatically reducing silk fibroin with palladium acetate. The catalyst exhibited an impressive chemical selectivity for the hydrogenation of olefins and azides, and the presence of MeOH was shown to aid palladium acetate reduction. Youyi Xia [108] extended this work by grafting a reductive water-soluble sulfonated polyaniline onto a silk fibroin. The synergistic effect of the water-soluble sulfonated polyaniline and tyrosine effectively improved the bearing capacity of modified silk fibroin, which was 30% higher than that of pure silk fibroin. Moreover, because of the high redox potential of the phenolic group, tyrosine can usually reduce the metal ion precursor and construct the metal–peptide hybrid structure through biomineralization [109]. Yong Zhou [110] used a protein in situ redox approach to create a novel core–shell nanostructured gold colloid–silk fibroin bioconjugate at room temperature. Au colloids with a high degree of stability were successfully transformed. Since then, protein in situ reductions and oxidation technology has been widely used in creating silk fibroin-based catalysts, using tyrosine residues to reduce metal particles so they may be stably loaded onto the silk threads. Another issue that must be considered at the outset of the catalyst is its recovery and reuse. In 2019, Parisa Akbarzadeh [111] prepared carbon nanotubes (CNT)-Fe_3_O_4_-fibroin-Ag nanocomposites using fibrin-functionalized magnetic CNT as green carriers for anchoring silver nanoparticles (Figure 4). The introduction of silk fibroin-based magnetic materials enabled continuous recovery and reuse of the catalyst for at least eight reactions without significantly degrading its catalytic performance.

**Figure 5:**
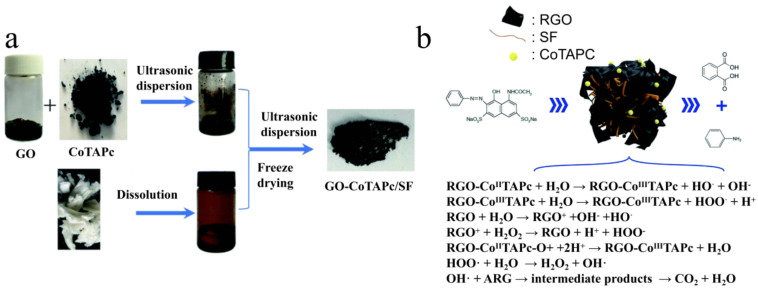
(**a**) Preparation process of GO-CoTAPc/SF gel, (**b**) degradation mechanism of ARG by RGO-CoTAPc/SF. Reprinted from Ref. [117] Copyright (2019) with permission from The Royal Society of Chemistry.

It is critical to logically construct the catalyst’s structure since it affects the coordination and arrangement of the surface atoms and the catalytic efficiency in general [112]. The 3D porous catalytic materials have a structure that successfully avoids metal particle agglomeration while maintaining the high surface area required for high catalytic effectiveness. The highly linked porous 3D networks will promote rapid electron and ion movement. Silk fibroin has a natural affinity for metals and has 18 amino acid structures and a typical natural protein hierarchical structure. Silk fibroin has a natural relationship with metals, and its 3D structure is thought to provide a suitable catalyst skeleton [113,114,115]. Kun-yuan [116] successfully prepared RSF hydrogel with magnetic catalysis loaded with Fe_3_O_4_ particles by chelating an interaction between RSF and Fe^2+^/Fe^3+^. Because of the strong interaction between Fe^2+^/Fe^3+^ and the silk fibroin molecular chains, RSF chelated more Fe_3_O_4_, and the Fe_3_O_4_ in the synthesized magnetic hydrogel had higher magnetic saturation. In addition, the RSF matrix was also used as a protective material for the Fe_3_O_4_ to isolate air and water, giving the Fe_3_O_4_ nanoparticles significant stability and maintaining peroxidase-like activity. Hui Ma [117] used RSF gel as the backbone of reduced GO (RGO) and cobalt tetra-amine phthalocyanine (CoTAPc) to prepare a gel catalytic system (RGO-CoTAPc/SF) (Figure 5). The RGO-CoTAPc/SF composite presented a thin-walled microporous interconnection network with a large specific surface area, and the CoTAPc molecules were homogeneously anchored on the RGO sheet. This structure provided abundant open edge sites and a large surface area, which improved the performance of the catalyst. The 3D porous RGO-CoTAPc/SF gel exhibited excellent catalytic degradation performance for acid red because of its high generation efficiency and the surface area of hydroxyl radicals (the dye degradation rate reaches 100%). In addition, the multiple amino acid residues and larger specific surface area of the RSF gel captured more acid red and reactive groups. In general, aerogel is a gel in which the hydrogel’s solvent is replaced by air, preserving the hydrogel’s 3D network structure while having ultra-low bulk density, high specific surface area, and high porosity [118]. Recently, Mitropoulos [119] created a silk fibroin composite aerogel with precious metal particles. The silk fibroin was cross-linked with ethanol, and the metal ions were reduced by sodium borohydride to balance the gel. After supercritical drying, the composite silk fibroin aerogel fiber with noble metal was obtained. These noble metal filament aerogel fibers have great development potential in noble metal catalysis, biosensors, and energy storage. Godiya [120] produced a low-cost, long-acting, and highly capable metal ion adsorption hydrogel made of silk fibroin and polyethyleneimine. The composite hydrogel also showed unusual catalytic activity because of the gradual adsorption and reduction of metal ions.

## 6. Catalysts by Using Collagen and Silk Fibroin as Precursors

Carbon materials have become effective catalyst supports because of their high conductivity, tunable microstructure, and excellent stability. However, traditional carbon compounds, such as graphene and CNT, have issues such as complicated preparation, low yield, and pollution [121,122]. As a result, it is vital to find green, efficient, low-cost preparation methods and renewable raw materials. Because it is abundant, renewable, non-toxic, and inexpensive, biomass is a promising carbon-rich precursor that excels in synthesizing functional carbon molecules [123]. In this section, we review the recent progress on catalysts by using collagen and silk fibroin as precursors.

### 6.1. Collagen-Derived Carbon Catalysts

Layered porous materials possess the pore size and topology favorable for catalytic reactions and high specific surface area. Layered porosity is necessary for novel porous carbon materials with controllable morphology, porosity, and structure, especially for carbon frameworks [124,125]. Because collagen fibers have a natural hierarchical structure and one-fourth of the nano-fibrils are interlaced, they provide a perfect 3D porous scaffold. Dehui Deng [126,127] synthesized mesoporous alumina and ZrO_2_ carbon fibers using collagen fibers as a template. The synthesized alumina fibers and ZrO_2_ carbon fibers successfully maintained the excellent structure of collagen fibers. Yang Liao [128] prepared a fibrous zirconium sulfate solid acid catalyst using collagen fibers as a template. The results indicated that, thanks to the excellent fiber morphology and porous structure of the SO_4_^2−^/ZrO_2_ catalyst, the conversion rate of the catalyst for the esterification reaction of n-butanol reached 99.0%. In addition, the catalyst could be reused six times without a significant loss of catalytic activity and good reusability. Gao Xiao [129,130] created mesoporous cerium sulfate-doped TiO_2_ (SO_4_^2−^-Ce_x_/TiO_2_) and cerium-doped TiO_2_ (Ce_x_/TiO_2_) using collagen fibers as a template to catalyze esterification and photodegradation processes, respectively (x represents the molar ratio of Ce^4+^ to Ti^4+^). Both retained the structure and morphology of natural collagen fibers and gave the catalyst a larger specific surface area, which improved the adsorption of the catalyst to the reactants. The conversion of SO_4_^2−^-Ce_0.02_/TiO_2_ was 99.9%, and the degradation of RhB by Ce_0.03_/TiO_2_ was 99.59%. Collagen-derived materials are also excellent carriers for the synthesis of electrocatalysts [33]. It is possible to give the carbon material generated from collagen a hierarchical porous structure as the catalyst’s carrier. Transport between reactants, electrolytes, and active sites during catalysis can be enhanced via hierarchical porous structures. Its numerous functional groups also serve as sources of N, P, O, and other atoms. Heteroatoms can alter the electrical and crystal structure of carbon-based materials, the surface electronic structure of catalysts, the production of new active sites, and the conductivity and electron donor properties, as well as the electrical and electronic properties of carbon-based materials themselves. This improves the electrocatalytic activity of the electrocatalysts. The electron donor’s conductivity and other qualities are improved by the crystal structure, which also alters the catalyst’s surface electronic structure, develops new active sites, and raises the conductivity [131,132,133]. Through the vacuum-assisted carbonization of collagen gel, Ying-Hui Lee created porous N-doped carbon nano-sheets and porous carbons [38,134]. Because of the N-doped structure, along with the high specific surface area and high porous structure, carbon nano-sheets and porous carbons demonstrated strong electrocatalytic activity for the ORR. The N-doped structure and oxygen-containing functional groups worked synergistically. Porous carbon outperformed the commercial platinum-based electrocatalysts in terms of hydrogen peroxide generation selectivity and electrocatalytic activity for the ORR. The electron-rich atoms contained in heteroatom-doped carbon materials resulted in a complete change in the material’s electronic properties. Because of the porous structure and abundant surface heteroatoms of the collagen-derived materials, they exhibited catalytic activity without introducing transition metals. Dan Luo prepared a carbonized self-assembled collagen scaffold as a hydrogen evolution reaction (HER) catalyst supported by Ru nanoparticles (Ru-CCS) [72]. The porous structure of the collagen scaffold provided sufficient sites for the loading and crystallization of Ru metal cations and dispersed them uniformly. The Ru-CCS composite increased carrier mobility and lowered the free energy of hydrogen chemisorption, resulting in high electrocatalytic activity. The TOF value of the Ru-CCS catalyst for the HER was 3.70 s^−1^, and the Faradaic efficiency was 96.4%. Wang used biomass collagen fibers as structural templates to synthesize bimetallic compounds embedded in N-doped carbon nanofibers (CNFs) [135]. The unique 1D fibrous structure of CNFs was conducive to the construction of high-efficiency conductive frameworks with high specific surface area, maximizing the exposure of active sites to improve electrical conductivity. When used as a separator modification material and a sulfur host simultaneously, the composite showed remarkable cycling stability with a capacity retention rate of 91.45% after 500 cycles. Gao Xiao [55] successfully prepared a unique Fe/copper phenol super structural building block with high electrocatalytic activity and reusability by the thermal carbonization of collagen/cellulose nanocrystals (Col/CNCs) of bimetallic polyphenol networks. The N self-doped carbon framework with a developed porous structure was coupled with copper ferrite nanoparticles with good crystallinity and uniform particle size. Because of the synergistic effect of Fe/Cu bimetallic and 3D interconnected hierarchical porous composite carbon aerogels, the as-prepared Fe_3_Cu_3_/C_ColCNCA_-800 °C (carbonization temperature 800 °C) achieved a limiting current density of 7.32 mA cm^−2^, far exceeding that of commercial Pt/C catalysts.

**Figure 6:**
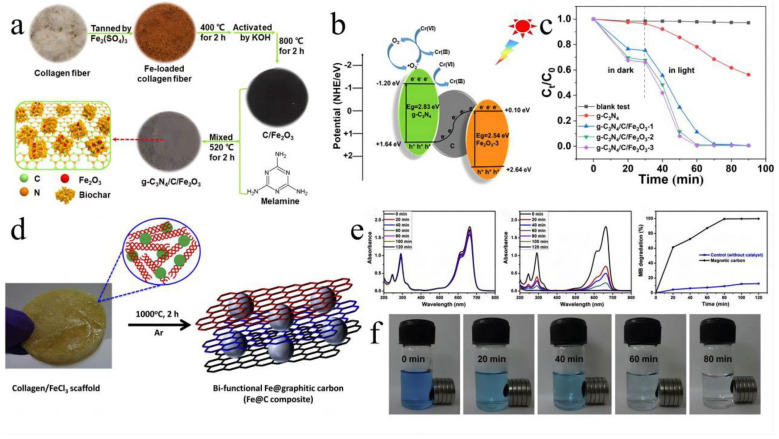
(**a**) Scheme for the fabrication of g−C_3_N_4_/C/Fe_2_O_3_, (**b**) schematic illustration of the reduction of Cr(VI) by g−C_3_N_4_/C/Fe_2_O_3_−3 under light irradiation, (**c**) Cr(VI) reduction efficiency of different photocatalysts. Reprinted from Ref. [136] Copyright (2021) with permission from American Chemical Society. (**d**) Schematic showing the synthesis of the Fe@C nanoparticles, (**e**) UV–Vis absorption spectra of MB degradation, (**f**) digital images showing the extent of the 10 ppm MB degradation within 80 min of sunlight irradiation and the magnetic separation of the catalyst from the treated solution. Reprinted from Ref. [138] Copyright (2019) with permission from Elsevier.

**Figure 7:**
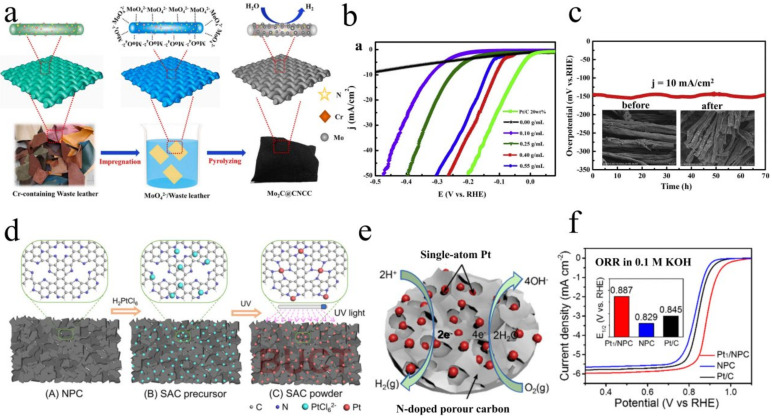
(**a**) Illustration of the simple synthesis process of an Mo_2_C@CNCC electrode from waste leather, (**b**) LSV curves of different Mo_2_C@CNCC catalysts in 1 M KOH solution, (**c**) chronopotentiometric measurements of the long-term stability of the Mo_2_C−0.4@CNCC catalyst at a current density of 10 mA/cm^2^, inset in (**f**) presents SEM images of Mo_2_C−0.4@CNCC catalyst before and after the long-term HER test. Reprinted from Ref. [139] Copyright (2020) with permission from Elsevier. (**d**) Schematic illustration of Pt_1_/NPC catalyst formation, (**e**) schematic diagram of catalytic HER, (**f**) the HER polarization curves of the Pt_1_/NPC, NPC, and 20% Pt/C catalysts recorded at a scan rate of 5 mV s^−1^ in N_2_-saturated 0.5 M H_2_SO_4_ solution; inset shows the enlarged curves at the potential onset region of the HER. Reprinted from Ref. [140] Copyright (2018) with permission from American Chemical Society.

Animal skin is a 3D network of collagen fibers with excellent elasticity and tension. For example, in pig skin, the collagen fibers and fiber bundles are mainly arranged in two directions, passing roughly obliquely between the epidermis and subcutis. This unique protein network structure provides a multi-level carbon skeleton for the catalyst. Zhao created a new graphitic carbon nitride (g−C_3_N_4_)/C/Fe_2_O_3_ photocatalyst using waste leather as a raw material (Figure 6a) [136]. Because C/Fe_2_O_3_ and g−C_3_N_4_ produce an indirect z-type heterojunction that increases the carrier separation efficiency, Cr(VI) can be decreased by up to 99.9% in 40 min (Figure 6c). Kuntal Chatterjee [137] reported a facile synthetic technique to convert goat skin waste into N-rich graphitic carbon materials with a nano-onion structure for a metal-free ORR catalyst. The main driving force for the ORR activity was the presence of pyridine N atoms, which, in addition to providing a net positive charge to neighboring carbon atoms, also served as active sites, facilitating oxygen adsorption and electron attraction at the anode. Good electrocatalytic performance was shown for the ORR in an alkaline medium. Compared with standard Pt/C catalysts, this catalyst exhibited superior long-term durability and higher methanol crossover performance. In 2019, Berhanu Telay Mekonnen [138] uniformly mixed FeCl_3_ solution with collagen from a waste leather solution and lyophilized it to obtain a FeCl_3_ collagen scaffold. Finally, the scaffold was carbonized at 1000 °C for 2 h to obtain Fe@C nanoparticles (Figure 6d). The synthesized Fe@C nanoparticles were spherical core–shell nanostructures with ferromagnetism at room temperature. They exhibited a 100% degradation rate for methylene blue under sunlight, and the catalyst recovery was simple (Figure 6e,f). Recently, Qiaoling Kang [139] soaked leather in (NH_4_)_6_Mo_7_O_24_⋅4H_2_O aqueous solution and calcined at high temperature to synthesize molybdenum carbide (Cr, N) co-doped carbon cloth (Mo_2_@CNCC) (Figure 7a). Mo_2_C nanoparticles were coupled and uniformly distributed in waste leather-derived carbon fibers, which was beneficial to the electrocatalytic performance. The Mo_2_C@CNCC catalyst was different from other MO_2_C HER electrocatalysts that require acidic media, and it exhibited excellent catalytic activity for HER in alkaline media (Figure 7b,c). With Mo_2_C@CNCC under the voltage of 2.0 V, the Faraday efficiency of the two-electrode device was close to 100%, and the volume ratio of H_2_ to O_2_ was 2.05:1. Furthermore, the Mo_2_C@CNCC catalyst was simple to utilize and could be directly employed as the working electrode.

Animal bones are mainly composed of hydroxyapatite and collagen, which have a natural 3D-ordered assembly. In porous carbon, collagen can supply carbon, N, and O, while hydroxyapatite serves as a natural template to regulate porosity. Wang reported the synthesis of N and O co-doped hierarchical porous carbon networks by pre-carbonization and potassium hydroxide activation using bovine bone as a precursor [141]. Liu [140] prepared single Pt atom catalysts using an N-doped porous carbon network (Pt_1_/NPC) from bovine bone (Figure 7d,e). The Pt atoms benefited from the high dispersion of Pt-N-C bond formation on the collagen-derived material carrier. The Pt_1_/NPC catalyst exhibited ultra-high electrocatalytic activity for the HER with an overpotential of 25 mV at 10 mA cm^−2^, a mass activity of 2.86 A mg^−1^ and good stability, which was significantly higher than that of the commercial Pt/C catalyst (Figure 7f). In addition, the Pt_1_/NPC catalyst also showed good catalytic activity for the ORR. Wang [142,143] created Co-N-C and Fe-N-C catalysts utilizing N-doped porous carbon derived from bovine bone as the support material for Co and Fe, which outperformed commercial Pt/C catalysts in terms of catalytic activity. The Fe-N-C catalyst, in particular, was based on Fe phthalocyanine/unsubstituted phthalocyanine (FePc/Pc) complexes as Fe precursors, with Pc molecules purposefully co-assembled with FePc molecules to offer steric sites for Fe demetallation. Simultaneously, the employment of in situ doped N atoms and a high number of micropores efficiently suppressed and stabilized the Fe atoms, allowing them to reach atomic-level dispersion over porous carbon. The catalytic activity of Fe-N-C catalysts for the ORR in acidic electrolytes was comparable to that of commercial Pt/C electrodes but with superior stability (7 mV negative shift after 3000 potential cycles). In alkaline media, the Fe–N–C catalyst exhibited better ORR activity and stability than commercial Pt/C electrodes (1 mV negative shift after 3000 potential cycles). In addition to the work described above, a part of the work of collagen-derived carbon in the field of electrocatalysis is summarized in Table 2. As shown in Table 2, the electrocatalysts prepared from animal bones and skins have excellent electrocatalytic performance, generally higher than current commercial catalysts. Moreover, compared with animal skin, animal bones are generally carbonized multiple times, which is more conducive to the formation of an excellent porous network structure. In addition to animal bones, animal waste such as discarded cowhide has been widely used to develop heterogeneous catalysts. Using these wastes in catalyst preparation can protect our environment and become the cheapest source of raw materials, reducing the cost of catalysts.

### 6.2. Silk Fibroin-Derived Carbon Catalysts

Silk has been created into an environmentally beneficial and much-used precursor because of the amino-rich and -sheet structure of silk fibroin, a protein biomaterial as plentiful as collagen [146,147]. As shown in Table 3, the structural tunability of silk fibroin-derived carbon makes it advantageous as an electrocatalyst. Moreover, the structures of the catalysts in the table are primarily 3D, further indicating that the porous network structure can significantly improve the catalytic performance of the catalyst. The construction of the 3D carbon skeleton can prevent the stacking and bundling of the corresponding low-dimensional structural units (powder and 1D), thereby shortening the diffusion distance of substances and charges, and greatly exposing the active sites, which can not only achieve easy recycling but also have excellent catalytic activity. The multi-layered structure of silk allows fine-tuning of silk functions by manipulating the molecular structure from nano to macro scales. Therefore, top-down and bottom-up approaches can be employed to generate silk fibroin-derived carbon catalysts with desired specific structures. The top-down approach obtains carbon-based catalysts by carbonizing the macrostructure of silk or the micro-nanofibrous structure exfoliated from silk. In Table 3, we summarize the structures of the silk fibroin-derived carbons and their electrocatalytic properties. Yongpeng Lei [148] ground the cocoons with melamine and g-C_3_N_4_, then heated the mixture at 600 °C for 1 h, and finally heated the sample at 900 °C for 1 h, demonstrating successful in situ growth of N-doped graphene (NG) on silk cocoon-derived interconnected carbon fibers (SCC_f_). The resulting catalyst (NG-SCC_f_) was used for the ORR and photocatalytic hydrogen production reactions. The onset potential of NG-SCC_f_ was close to that of commercial Pt/C, and the methanol tolerance and stability in the alkaline medium were better than those of Pt/C. In the photocatalytic reaction, the photocatalytic H_2_ yield of NG-SCC_f_ was 66.0 μmol·h^−1^·g^−1^. The excellent catalytic activity was attributed to the introduction of more active sites by NG and the 3D network structure, which increased the electron transfer efficiency. The β-sheet structure of proteins in silkworm cocoons can be transformed into N-doped conjugated sp2 hybrid hexagonal carbon structures through pre-carbonization at lower temperatures. Yixuan Wang [149] synthesized S- and N-doped micro-mesoporous carbons (SNC-x) from silk cocoons as predecessors. At 800 °C, the SNC-x formed nanoparticles with a diameter of 800–500 nm and interconnected pores to form a dichasial architecture. This cramped and loose aggregation provided SNC-x with a unique layered porous structure. The unique architecture of SNC-x provided abundant active sites, greatly promoted the transportation of reactants to active sites, and enhanced ORR activity. The initial potential of SNC-x in the ORR was 0.853 v. Mengjie Chen [150] reported the preparation of carbon-based electrocatalysts with inherent defects after the two-step carbonization of silk cocoons. He first performed pre-carbonization at 350 °C, and then, at 1000 °C, the pre-carbonized material was further activated in the presence of zinc chloride. The activation step created pores and inherent defects in the carbon material with a larger surface area. The resulting electrocatalysts catalyzed the reduction of carbon dioxide to CO with a Faradaic efficiency of 89% and selectivity was maintained for 10 days. Furthermore, this demonstrated that inherent defects in the carbon matrix were the main reaction sites, especially those containing pentagons. The carbon dioxide reduction rate-determining step was the direct electron transfer to carbon dioxide rather than the proton-coupled electron transfer. Silk has a natural fibrous structure, and fibers are 1D materials, which can effectively overcome the problem of material agglomeration by forming a porous structure, which is also beneficial to mass diffusion and ion transport in the catalytic process. Tomoya Iwazaki [151] prepared a carbon-based metal-free catalyst with high catalytic activity for the ORR by carbonizing silk fibroin. After carbonization at 500 °C, the silk fibroin carbon was activated with steam to increase the surface area of the activated carbon so that it had a high catalytic activity for the ORR.

**Figure 8:**
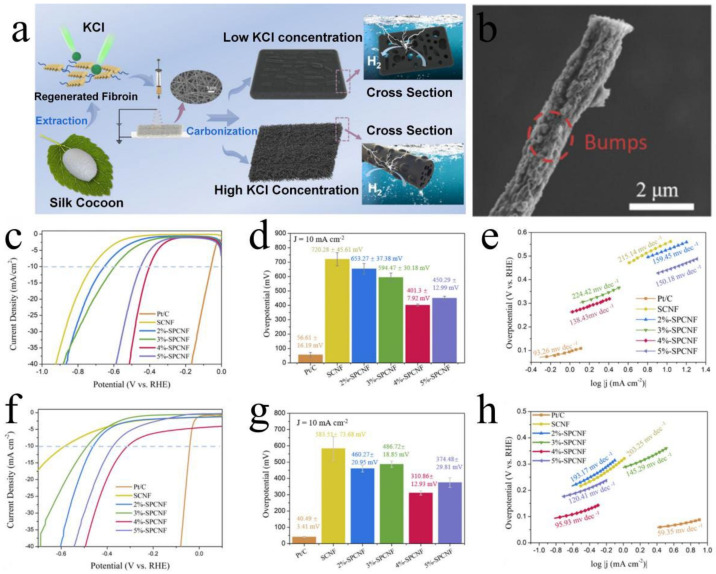
(**a**) Schematic diagram of porous CNFs prepared by electrospinning and HER. (**b**) SEM image of 4%−SPCNF single fiber. Acidic HER performance for (**c**) LSV curves of (**d**) bar chart of overpotential values, (**e**) Tafel slopes. Alkaline HER performance for (**f**) LSV curves of (**g**) bar chart of overpotential values, (**h**) Tafel slopes. Reprinted from Ref. [152] Copyright (2022) with permission from American Chemical Society.

**Figure 9:**
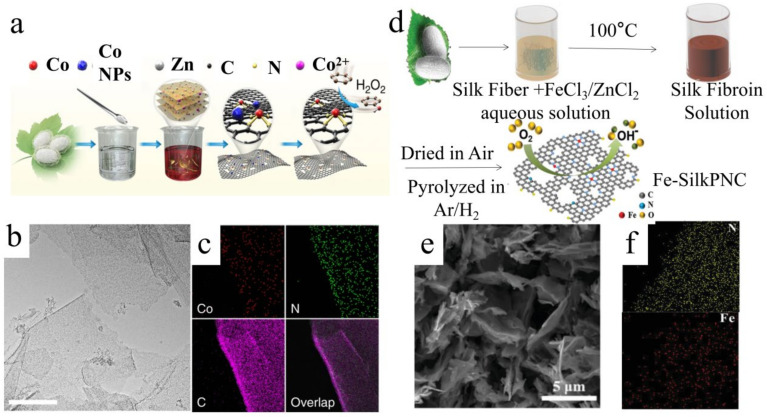
(**a**) Preparation process of a Co-ISA/CNS catalyst [161], (**b**) TEM image of Co−ISA/CNS, (**c**) EDX elemental mapping of CoISA/CNS. Reprinted from Ref [161] Copyright (2018) with permission from Springer Nature. (**d**) The reparation process of Fe-SilkPNC, (**e**) TEM image of Fe-SilkPNC, (**f**) EDX elemental mapping of Fe-N_x_-C. Reprinted from Ref. [153] Copyright (2019) with permission from Wiley.

Silk fibroin can be dissolved and regenerated in a high-concentration transition metal salt solution, after which it can self-assemble into the desired structure [158]. Therefore, the bottom-up method for producing silk fibroin-derived carbon-based catalysts is also widely used. Hongzhe He [152] first dissolved degummed silk in a CaCl_2_/CH_3_CH_2_OH/H_2_O ternary solvent system and then self-assembled to form RSF. The electrospinning solution of silk fibroin nanofibers was prepared by dissolving RSF fibroin in anhydrous formic acid. Nanoscale silk fibers were prepared by electrospinning, and then the silk fibers were activated and carbonized to obtain silk fibroin-derived porous CNFs (SPCNF) (Figure 8a). After chemical activation by KCl, the CNFs formed a hierarchical porous structure, and the porous structure and fiber morphology exposed more active sites (Figure 8b). Compared with the direct carbonization of micron-sized silk, the CNFs obtained by electrospinning had a larger specific surface area and higher porosity. The 4%−SPCNF (the mass fraction of KCl in the spinning solution is 4%) exhibited superior catalytic activity for the HER under acidic and basic conditions. The overpotential and Tafel slope of 4%-SPCNF under acidic conditions were 310.86 ± 12.93 mV and 95.93 mV dec^−1^ (Figure 8c–e), and the overpotential and Tafel slope under alkaline conditions were 401.3 ± 7.92 mV and 138.43 mV dec^−1^ (Figure 8f–h), respectively. Compared with metal-based catalysts, especially precious metal catalysts, carbon-based metal-free catalysts save costs and are expected to become excellent substitutes for precious metals or transition metal catalysts [159,160]. Youqi Zhu [161] used a sodium carbonate aqueous solution to remove the gelatinous silk protein from silk and then obtained silk fibroin. The silk fibroin was dissolved in a mixed solution of cobalt chloride and zinc chloride to obtain silk fibroin solution. In particular, traditional whole water extraction of silk fibroin converted natural silk fibers into a lamellar structure containing β-sheet crystals. The RSF was carbonized to synthesize metal single-site catalysts embedded in ultra-thin two-dimensional (2D) porous N-doped carbon nano-sheets (M-ISA/CNS, M = Fe, Co, Ni) (Figure 9a). The M-ISA/CNS had an enormous specific surface area because of its 2D sheet structure and single metal atoms (Figure 9b,c). The O=Co=O central intermediate generated on a single Co site exhibited excellent catalysis activity. The Co-ISA/CNS catalyst had high-activity characteristics for the direct catalytic oxidation of benzene to phenol at room temperature, and the selectivity to phenol reached 97% within 1 h. Chunya Wang [153] exploited silk fibroin dissolution and regeneration to create 2D porous carbon nano-sheets as carriers for single-atom Fe (Fe-N_x_-C) (Figure 9d). The active sites of Fe-N_x_-C featured large 2D porous structures. The single-atom form of Fe was consistently distributed across the carbon nano-sheets (Figure 9e,f), demonstrating high OER catalytic activity. In addition to preparing 2D structures, silk fibroin is a support material for preparing 3D structural materials [162,163]. In 2018, Taek-Seung Kim [164] reported RSF as a 3D scaffold and carbon source to synthesize a 3D-patterned cobalt phosphide/carbon structure catalyst composed of nano-sheets connected by silk fibrin. A flake-like Co(OH)_2_ precursor was created when 0.5 wt% RSF experienced a hydrothermal reaction in an alkaline solution. A Co pattern was formed by constructing each Co(OH)_2_ nano-sheet precursor after a subsequent hydrothermal process. Because of the unusual structure, its 3D structure and carbon matrix boosted charge transport and provided more active sites, resulting in increased catalytic activity and better stability and reusability. In the same year, Changqing Li [165] reported that RSF was used as a scaffold to obtain a heteroatom (N, S, and Fe) ternary-doped porous carbon aerogel. Silk fibroin amino acids acted as ligands, forming FeNx coordination complexes with Fe ions, increasing the number of active sites. The heteroatoms that entered the sp2 carbon framework increased the catalyst’s defects and improved ORR activity. The N and S heteroatom doping and porous structure worked together to improve ORR activity.

## 7. Summary and Outlook

Here, we reviewed applications and developments of catalysts with collagen and silk fibroin as carriers and precursors. Collagen-containing biological tissues or silk fibers can be converted to porous carbon materials with desirable heteroatoms (N, O, and S heteroatoms) as catalysts. Owing to the hierarchical structural characteristics of natural collagen and silk fibroin, porous carbons with different porosities (macroporous, microporous, and mesoporous) and morphologies (1D nanofiber, 2D nano-sheet, and 3D network) can be designed and synthesized. In addition, the appealing properties of collagen and silk fibroin, including their biocompatibility and excellent mechanical properties, make them good substrates and candidates for constructing catalysts. Therefore, their renewable large-scale global supply and environmental benignity render collagen and silk fibroin as alternatives to traditional inorganic materials and non-biodegradable synthetic polymers in the sustainable development of catalysts.

The past few years have witnessed the emergence of collagen- and silk fibroin-based catalysis applications. However, their development remains at a rudimentary stage, and this research field is faced with challenges. Self-doping or external-doping porous carbon materials for catalysis application have been developed through carbonization and activation methods. Systematic strategies must be designed for the synthesis of collagen-derived and silk fibroin porous carbon materials with controllable morphology, pore size, porosity, and even heteroatomic compositions. Moreover, large-scale production of collagen and silk fibroin materials is difficult because of the tedious processing required for protein extraction and dissolution, as well as maintaining the storage conditions. Therefore, new improved processing methods are expected to be proposed.

## Figures and Tables

**Table 1:** Polyphenol cross-linking agents used to construct collagen-based catalysts.

Metal Nanoparticles	Cross-Linking Agent	Type of Catalytic Reaction	Catalytic Efficiency	References
Pd	EGCG	Hydrogenation of nitrobenzene and its derivatives	Nitrobenzene conversion rate 98%, selectivity 99%	[89]
Pd	EGCG	Hydrogenation of allyl alcohol	Conversion rate 99.8%, selectivity 89.04%	[19]
Pd	EGCG	Stille coupling reactions	The yield of static coupling of aryl iodide with vinylbutane is 90%	[99]
Pt	EGCG	Hydrogenation of typical olefins	Conversion rate > 99.5% Selectivity > 99%	[100]
Pd	Black wattle tannin	Hydrogenation of quinoline	Conversion rate 99.3%, selectivity 100%	[90]
PtCo*x*	BT	Hydrogenation of cinnamaldehyde	The conversion of cinnamaldehyde was 93.56%	[101]
Au@Pd	BT	Liquid-phase hydrogenation of cyclohexene	The conversion was 92.70%	[102]
Ru	BT	Hydrolyze ammonia borane	TOF was as high as 322 mol_H2_ mol^−1^min^−1^ and Ea was as low as 32.41 kJ mol^−1^ for AB hydrolysis	[91]

**Table 2:** Collagen-derived carbon catalysts for electrocatalysts.

Raw Materials	Catalysis	Carbonization Temperature and Time	Reaction	Medium	Activity	Ref.
Cattle bone	SA-Fe-HPC	400 °C; 2 h 900 °C; 1 h	ORR	Acidic electrolyte	E_1/2_ = 0.81 V, J_d_ = 5.5 mA cm^−2^	[143]
				Alkaline electrolytes	E_1/2_ = 0.63 V, J_d_ = 2.8 mA cm^−2^	
Pig bone	Fe-N-HPC-AH	400 °C; 3 h, 800 °C; 1 h, 800 °C; 2 h	ORR	Alkaline electrolyte	E_onset_ was 0.97 V, E_1/2_ was 0.870 V	[144]
Cattle bones	Co-N-HPC	400 °C; 3 h, 850 °C; 1 h 800 °C; 2 h	ORR	Alkaline electrolyte	E_1/2_ = 0.835 V J_k_ = 20.4 mA cm^−2^	[142]
Cowhide	FeCu/C_Col-CNCA_	800 °C; 5 h	ORR	Alkaline electrolyte	The current limit density reached 7.32 mA cm^−2^	[55]
Cattle bone	Pt_1_/NPC	400 °C; 3 h 800 °C; 1 h 1000 °C; 3 h	HER	Acidic electrolyte	At −10 mA cm^−2^, the potential is −0.025 V. The TOF was 2.93 s^−1^	[140]
			ORR	Alkaline electrolytes	E_1/2_ = 0.835 V The largest J_k_ was 3.23 mA cm^−2^	
Cr-containing leather	Cr/CF600	600 °C; 3 h	ORR	Alkaline electrolytes	The oORR yields H_2_O_2_ with selectivity ~86% at 0.62 V	[145]
Rat tail tropocollagen	Ru-CCS	800 °C; 2 h	HER	Acidic electrolyte	E_onset_ was 11.0 mV The TOF was 3.70 s^−1^ at an overpotential of 50 mV,	[72]
Goat skin	750-8 N-CNO	750 °C; 8 h	ORR	Alkaline electrolytes	50 mV onset potential at 10 mA cm^−2^	[138]
Waste leather	Mo_2_C@CNCC	900 °C; 3 h	HER	Alkaline electrolytes	272 mV overpotential at 10 mA cm^−2^	[139]

**Table 3:** Silk-derived carbon catalysts for electrocatalysts.

Catalysis	Structure	Carbonization Temperature and Time	Reaction	Medium	Activity	Ref.
A-350–1000	Particles	350 °C; 1 h 1000 °C; 1 h	CO_2_ reduction reaction	Acidic electrolyte	The Faradaic efficiency was 89% and maintained good selectivity for about 10 days.	[150]
4%-SPCNF	1D	900 °C; 4 h	HER	Acidic electrolyte	The overpotential was 310.86 ± 12.93 mV and Tafel slope was 95.93 mV dec^−1^	[152]
				Alkaline electrolyte	The overpotential was 401.3 ± 7.92 mV and Tafel slope was 138.43 mV dec^−1^	
Fe–N_x_–C	2D	220 °C 45 min, 320 °C; 2.5 h, 900 °C; 2 h	ORR	Alkaline media	Half-wave potential (E_1/2_) was 0.853 V, remarkable stability with only 11 mV loss in E_1/2_ after 30,000 cycles	[153]
CoW@ACSF	Particles	900 °C; --	HER	Acidic electrolyte	The overpotential was 138.42 ± 10.39 mV at 10 mA cm^−2^	[154]
			ORR	Alkaline electrolytes	The overpotential was 492.05 ± 19.04 mV at 10 mA cm^−2^	
SF-Cu/CA	3D	100 °C; 30 min, 225 °C; 2 h, 800 °C; 2 h	CO_2_ reduction reaction	——	The current density was 29.4 mA cm^−2^ and Faraday efficiency was 83.06%	[155]
NFe0.5-C	3D	900 °C; 2 h	ORR	Acidic electrolyte	The positive initial potential was 0.274 V and half-wave potential 0.095 V.	[156]
SFCA-NiCo	3D	225 °C; 30 min 800 °C; 2 h	HER	Alkaline electrolytes	E_onset_ was 52.0 mV 179 mV overpotential at 10 mA cm^−2^	[157]
CA-NiCoFe-600	3D	600 °C; 2 h	OER	——	321 mV overpotential at 10 mA cm^−2^ and the Tafel slope was 42 mV dec^−1^	[39]

## Data Availability

Not applicable.
